# Serial Changes in Exercise Capacity, NT-proBNP, and Adiponectin in Patients with Acute Coronary Syndrome before and after Phase II Rehabilitation as well as at the 12-Month Follow-Up

**DOI:** 10.1155/2022/6538296

**Published:** 2022-01-24

**Authors:** Hong Jin, Yuefei Liu, Bernd Schweikert, Harry Hahman, Lei Wang, Armin Imhof, Rainer Muche, Wolfgang König, Jürgen M. Steinacker

**Affiliations:** ^1^Division of Sports and Rehabilitation Medicine, Department of Cardiology, University of Ulm, Ulm, Germany; ^2^Department of Cardiology, Southeast University, Nanjing, China; ^3^Institute of Health Economics and Health Care Management, Helmholtz Center Munich, Germany; ^4^Schwabenland Hospital, Isny-Neutrauchburg, Germany; ^5^Department of Chinese Medicine Rehabilitation, Nanjing University of Chinese Medicine, Nanjing, China; ^6^Department of Cardiology, University of Ulm, Ulm, Germany; ^7^Institute of Biometrics, Ulm University, Ulm, Germany

## Abstract

**Background:**

Acute coronary syndrome (ACS) causes pathophysiological changes in exercise capacity, N-terminal part of pro-brain natriuretic peptide (NT-proBNP), and adiponectin that impact the course of coronary artery disease and clinical outcomes after cardiac rehabilitation (CR). However, the serial changes and the relationship between the changes in these parameters for a prolonged term remain uninvestigated.

**Methods:**

Eighty-one patients with ACS underwent a three- or four-week CR program after acute care and were followed up for 12 months. Exercise capacity on a cycle ergometer and blood levels of NT-proBNP and adiponectin were determined before and after CR as well as at the 12-month follow-up.

**Results:**

Exercise capacity increased from 100 watts (in median) before CR to 138 watts after CR and 150 watts at 12 months. The NT-proBNP level (526 pg/ml before CR) remained almost unchanged after CR (557 pg/ml) and then decreased at 12 months (173 pg/ml). The adiponectin level (14.5 *µ*g/ml before CR) increased after CR (16.0 *µ*g/ml) and at 12 months (17.2 *µ*g/ml). There was no significant correlation among the changes in these parameters at each observation time point.

**Conclusion:**

During the observation period from before CR to the 12-month follow-up, exercise capacity, NT-proBNP, and adiponectin underwent significant changes; however, these changes were independent from each other and not correlated linearly, and they provide complementary information in clinical practice. Thus, all these parameters should be included and determined at different time points for a prolonged period of time.

## 1. Introduction

Acute coronary syndrome (ACS), composed of unstable angina pectoris and myocardial infarction with or without ST segment elevation, is a common, critical event that threatens public health and jeopardizes work ability and quality of life [1]. Cardiac rehabilitation (CR) has been proven to be an important part of integrated medical care. Currently, CR is extensively implemented in the care of ACS patients, and it is well documented that CR exerts a significant beneficial effect on the clinical outcomes of patients with ACS [2].

Although there are a number of well-established parameters available for evaluating the effect of CR [3], most studies on the CR effect have reported one or a few parameters at a single time point or over a relatively short period of time, and there seems to be a lack of studies on a prolonged follow-up after phase II CR with different key parameters. ACS and CR bring about a variety of physiological changes in terms of myocardial remodeling, and these changes can be reflected by different parameters [4]. Exercise capacity is regarded as the most important parameter for the assessment of the CR effect [2], and numerous studies have shown that CR significantly improves exercise capacity [5, 6]. On the other hand, reduced physical capacity is an important risk factor for coronary artery disease [7]. Therefore, monitoring changes in exercise capacity in patients with ACS is meaningful for long-term follow-up [8].

During the myocardial remodeling process, the N-terminal part of pro-brain natriuretic peptide (NT-proBNP) undergoes changes that are associated with the severity of myocardial injury as well as left ventricular (LV) dysfunction [9]; therefore, NT-proBNP serves as an important parameter for monitoring subsequent LV remodeling and clinical outcomes in patients with ACS [10]. A previous study showed impaired exercise capacity along with an increase in NT-proBNP [11] in heart failure [12].

It is known that a variety of factors, especially metabolic factors, can impact the long-term outcome of ACS, and the adipocytokine adiponectin is regarded as an important cardiovascular protective agent [13]. Epidemiologic data have shown that a lowered adiponectin level is related to the progression of ACS [14]. Hence, for the assessment of the long-term CR effect on ACS, monitoring adiponectin levels is meaningful.

There are different studies dealing with changes in exercise capacity, NT-proBNP, and adiponectin levels in patients with ACS; however, the relationship among these parameters during a prolonged follow-up was not reported. Because these parameters are different in terms of their regulation and dynamic changes in patients with ACS and provide information from different clinical aspects, investigating the serial changes in these parameters, especially their relationship for a prolonged period, is of clinical significance. The present study sought to investigate the changes in exercise capacity, NT-proBNP, and adiponectin levels in patients with ACS before and after CR as well as at the 12-month follow-up while paying special attention to the relationship among these parameters.

## 2. Materials and Methods

### 2.1. Subjects

All patients were enrolled through the Department of Cardiology of University of Ulm and underwent percutaneous coronary intervention in acute care before CR. Patients treated with CABG from the Department of Cardiac Surgery were not included in the study. ACS was diagnosed according to the standards of the European Society of Cardiology [15]. The criteria for patient recruitment in this study were as follows: (1) confirmed coronary artery disease by angiographic imaging; (2) ACS was confirmed by typical chest pain, electrocardiography (ECG) findings, and subsequent elevation of cardiac enzymes (troponin *T* and serum creatine kinase-MB), including ST-elevation myocardial infarction, non-ST-elevation myocardial infarction, or unstable angina; (3) accomplishment of acute treatment; and (4) consent to CR and subsequent follow-up at 12 months. Patients with the following conditions were excluded from the study: (1) decompensated cardiac conditions such as dyspnea at rest and pronounced congestion such as obvious edema in the lower limbs; (2) unfinished acute care, hypertrophic cardiomyopathy, valvular disease requiring surgery, pericarditis, and severe renal dysfunction (i.e., creatinine>2.5 mg/ml); and (3) patients suffering from any noncardiac disease that would impede participation over the full length of the study period.

A total of 163 patients were initially enrolled in the study finally, after exclusion of drop-out cases.

**Figure 1:**
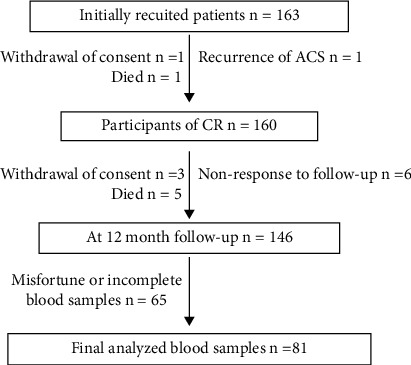
Flowchart of participant recruitment in the study.

The data from 81 patients were taken for the present study (Figure 1). The basic clinical data are summarized in Table 1.

This study was approved by the Ethics Committee of the Medical Faculty of the University of Ulm, and all the subjects gave their informed consent prior to the study.

### 2.2. Study Protocol

Upon the onset of ACS, all patients were acutely treated in the Department of Cardiology of University Hospital Ulm (Germany). After acute care, the patients were introduced to phase II CR according to the standards of the German Society of Prevention and Rehabilitation. After CR, the subjects were followed up for 12 months, and during the follow-up period, the patients were advised to join the phase III CR program. The measures performed in the phase III CR program were recommended by the American College of Sports Medicine and American Heart Association [16], and all the patients actively participated in the heart groups where one session of sport was performed weekly under professional guidance of a therapist and supervised by physicians from the study group. The weekly physical activity at moderate intensity of the patients was approximately 4 hours each week during the phase III CR. The clinical and laboratory data used in the study were collected before and after phase II CR and at 12 months after CR.

### 2.3. Phase II CR Program

A phase II CR program was performed according to the standards of the German Society of Prevention and Rehabilitation [17]. The major program elements consisted of patient education, social-medical and psychological support, nutritional intervention, physical training, and medication and lasted three to four weeks with five sessions each week. The daily program was approximately 6 hours with corresponding breaks between program elements and had approximately three hours of physical training, including endurance training on cycle ergometers, strength training on training devices, gymnastics, and coordination exercises. The exercise intensity of endurance training on ergometry was set individually at 50–60% of maximal workload determined by exercise testing on a symptom-limited cycle ergometer. During the phase II CR, the patients were not asked to do additional physical training at home separately from the CR program.

### 2.4. Clinical Investigation

Clinical examination was performed according to the standards of the German Society of Cardiology. The LV function was assessed through routine clinical echocardiographic examination. Generally, all patients showed diastolic dysfunction with a disturbed E/A ratio by pulsed-wave Doppler and/or E/*E*′ by tissue Doppler (the data were not completely analyzed and are not shown here). Therefore, the major parameter, i.e., EF was taken to analyze LV dysfunction. LV function was assessed by standard routine echocardiography. LV dysfunction was classified when the ejection fraction was <50% [18]. Baseline information on sociodemographic variables, smoking habits, medication, and history of disease were collected by physicians through a standardized face-to-face interview and questionnaire. Quality of life was assessed by using a questionnaire, the EQ-5D (European Quality of Life 5 Dimensions) [19]. The EQ-5D assesses the current health state in five aspects: mobility, self-care, usual activities, pain/discomfort, and anxiety/depression. The categories of the response offer three levels: no problems (score of 1), moderate problems (2), and extreme problems (3). The EQ-5D score (0 for the worst health and 100 for the best health) was taken for the study analysis (Table 1).

### 2.5. Determination of the Study Parameters

The exercise capacity was determined by symptom-limited exercise testing on a cycle ergometer. The standard protocol for ergometry was adopted from the guidelines of the German Society of Cardiology, beginning at 25 watts with 25 watt increments every 2 minutes until subjective exhaustion or criteria for termination of a stress test with continuous monitoring by a 12-lead ECG [20]. Blood pressure (Riva Rocci) and heart rate were recorded at rest, at the end of each workload and 1, 3, and 5 minutes during recovery from the stress test. Exercise capacity was expressed by the relative maximal workload and calculated as follows: relative maximal workload (watts) = workload before last step + (seconds at the last step/120) × 25.

For determination of NT-proBNP and adiponectin levels, fasting venous blood samples were drawn into tubes containing ethylenediamine tetraacetic acid under identical conditions. No exercise training or testing was performed within 24 hours prior to blood sampling. The blood plasma was collected and stored at −70°C. NT-proBNP was determined using an ELISA kit (Roche Diagnostics, Mannheim, Germany), and the total adiponectin concentration was determined by using an ELISA kit (Assaypro, Missouri, USA).

### 2.6. Statistical Analysis

All data (except for the clinical chemical data that were expressed as mean ± SD) were expressed as the median (interquartile range) for skewed continuous variables. NT-proBNP, adiponectin, and workload were logarithmically transformed to normalize their distribution for univariate analysis. The changes in a parameter with a series of measurements were examined by univariate analysis *with a general linear model*. Comparisons between groups were performed by the *Mann–Whitney U* test. S*pearman*'s or *Pearson*'s correlation method was used for correlation analysis. Statistical analysis was performed using SPSS software 25 (SPSS Inc., Chicago, USA). All tests were two-sided, and a difference was assumed to be statistically significant at *P* < 0.05.

## 3. Results

The basic data of the subjects, including anthropometry, cardiovascular risk factors, treatment, LV function, and quality of life (scores) are shown in Table 1. Overall, there was an improvement in cardiovascular risk factors, including smoking and cholesterol levels, over the observation period but no change in the body mass index. The number of patients with LV dysfunction did not change after CR but decreased at 12 months.

### 3.1. Exercise Capacity

**Figure 2:**
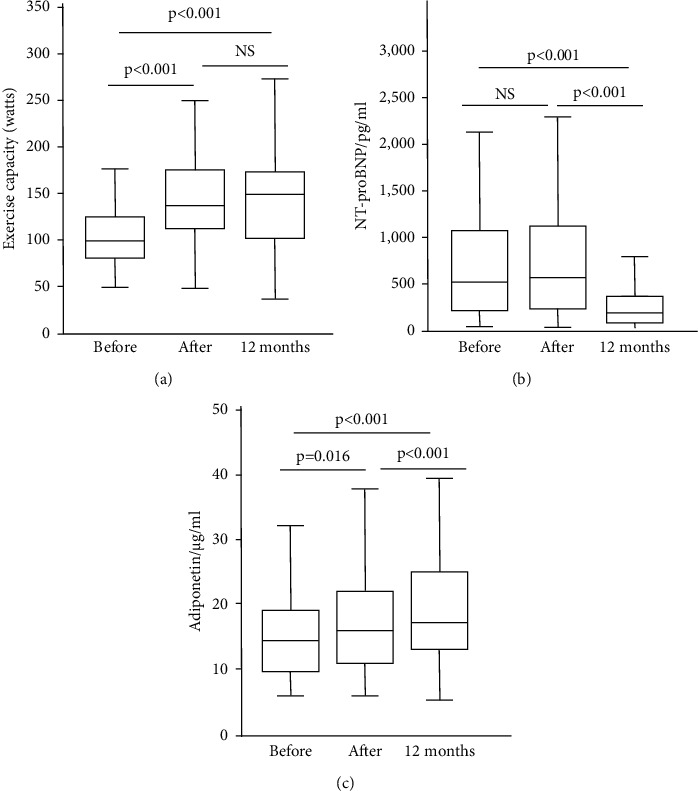
Serial changes in exercise capacity (a), blood level of N-terminal part of the pro-brain natriuretic peptide (NT-proBNP) (b), and adiponectin (c) before and after cardiac rehabilitation and at 12-month follow-up. The data are expressed in median with quartiles and the minimal and maximum.

**Figure 3:**
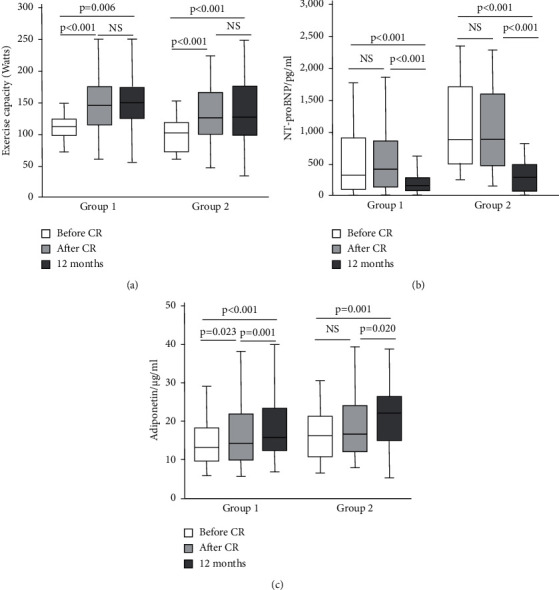
Serial changes in exercise capacity (a), blood level of N-terminal part of the pro-brain natriuretic peptide (NT-proBNP) (b), and adiponectin (c) before and after cardiac rehabilitation as well as at 12-month follow-up in patients divided to group 1 (*n* = 54, normal left ventricular function) and group 2 (*n* = 27, impaired left ventricular function). The data are expressed in median with quartiles and the minimal and maximum.

The exercise capacity data are depicted in Figure 2(a). In comparison with that before CR, the exercise capacity increased significantly after CR (*P* < 0.01) and at the 12-month follow-up (*P* < 0.01). This increase was found in both subjects with normal LV function and those with LV dysfunction (Figure 3(a)), although the exercise capacity in patients with LV dysfunction was generally lower than that in patients with normal LV function. Although the increase in exercise capacity in the normal LV function group was slightly higher than that in the LV dysfunction group (by 8 watts), the difference was not statistically significant.

### 3.2. NT-proBNP Level

Compared to that before CR, the NT-proBNP level did not change after CR but significantly decreased at 12 months (*P* < 0.01) (Figure 2(b)). In patients with LV dysfunction, the NT-proBNP level was higher than that in patients with normal LV function (Figure 3(b)). NT-proBNP levels decreased in all patients at 12-month follow-up (*P* < 0.01). Furthermore, the decrease in NT-proBNP levels was greater in patients with LV dysfunction than in those with preserved LV function (*P* < 0.001).

### 3.3. Adiponectin Level

The adiponectin level data are shown in Figure 2(c). Compared to that before CR, the adiponectin level increased significantly after CR (*P* < 0.05) and showed a further augmentation at the 12-month follow-up (*P* < 0.01). There was a tendency for adiponectin levels to be higher in the group with LV dysfunction than in the group with normal LV function, but the difference was not statistically significant (Figure 3(c)). The changes in adiponectin levels after CR were different between the two groups with regard to LV function: in the group with normal LV function, adiponectin levels increased after CR (*P* < 0.05), whereas they did not change significantly in the group with LV dysfunction. Both groups showed an increase at 12 months (*P* < 0.05).

### 3.4. Relationship of the Changes in Exercise Capacity, NT-proBNP, and Adiponectin

**Figure 4:**
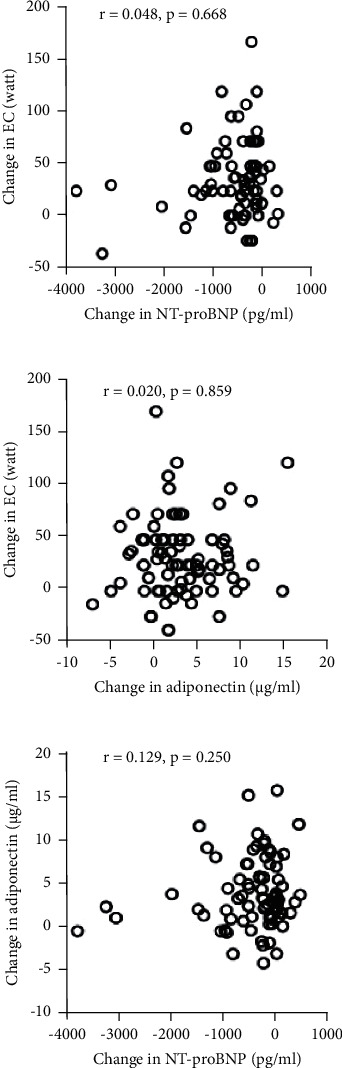
Relation between changes in N-terminal part of the pro-brain natriuretic peptide (NT-proBNP), adiponectin, and exercise capacity (EC) from baseline to 12 months.

No statistically significant correlation was found between changes in NT-proBNP, adiponectin, and exercise capacity from baseline to the 12-month follow-up (Figure 4). In addition, the changes in all three parameters before CR and after CR and between after CR and at the 12-month follow-up were not correlated.

**Figure 5:**
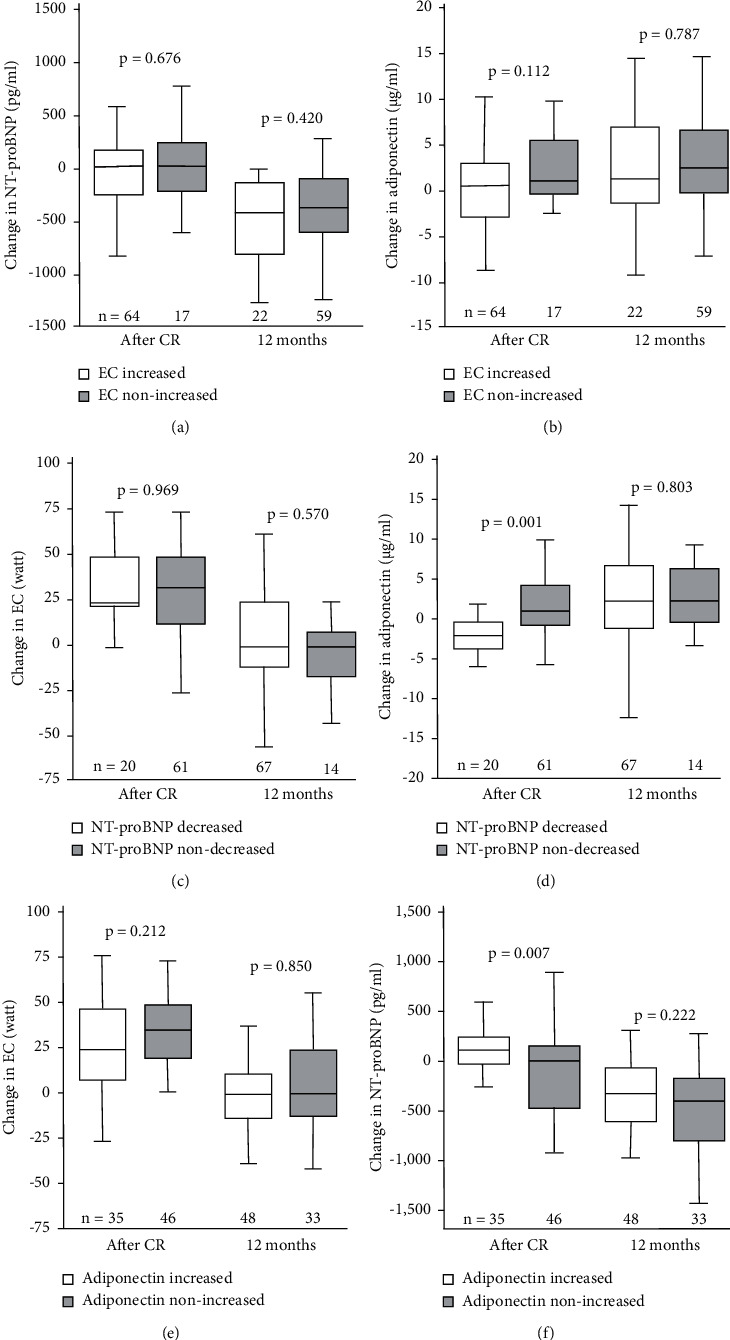
Structured analyses on changes in N-terminal part of the pro-brain natriuretic peptide (NT-proBNP), adiponectin, and exercise capacity (EC) during cardiac rehabilitation (CR) and at 12-month follow-up.

We performed further analysis based on changes in each parameter. According to changes in exercise capacity (increased or nonincreased), there was no significant difference in NT-proBNP after CR or at the 12-month follow-up (Figure 5(a)), and a similar result was found for adiponectin levels (Figure 5(b)). Based on the changes in NT-proBNP (decreased or nondecreased), no significant difference was found in exercise capacity after CR or at the 12-month follow-up (Figure 5(c)). The patients in the NT-proBNP nondecreased group showed higher adiponectin levels than those in the NT-proBNP decreased group during the CR period (from before to after CR), but there was no difference at 12 months (Figure 5(d)). According to changes in adiponectin, no significant difference between the increased and nonincreased groups was found in exercise capacity (Figure 5(e)) or in NT-proBNP (Figure 5(f)).

## 4. Discussion

ACS is a critical event that significantly threatens public health, and phase II cardiac rehabilitation is of clinical importance for the outcome of patients with ACS. Exercise capacity, NT-proBNP, and adiponectin are proven meaningful parameters that are commonly used for evaluating the effects of secondary prevention. However, to date, most of the studies dealt with only one of these parameters or only at a single time point, and few studies have investigated serial changes in all these parameters. Therefore, we investigated the characteristic changes in these parameters over a prolonged follow-up period and specifically analyzed their relationship.

Improving exercise capacity is of clinical relevance since physical performance serves as one of the most important predictors for the outcome of patients suffering from ACS [21]. It is closely related to quality of life [22] and a determinant for preserving work ability [23]. In the present study, the exercise capacity in the patients with ACS was clearly reduced before rehabilitation and increased significantly after CR (Figure 2(a)), which is comparable to the result reported by Giallauria et al. [24]. In the follow-up period, there was a further increase in exercise capacity, although this change was not statistically significant in comparison with that after CR. This means that the measures of secondary prevention could further improve or at least preserve the beneficial effect of CR for a prolonged period. LV function is known as a major determinant of exercise capacity in patients with heart disease [25], and LV dysfunction impairs physical ability [26]. In our present study, exercise capacity in patients with LV dysfunction was clearly lower than that in patients with preserved LV function (Figure 3(a)). However, exercise capacity increased after CR not only in patients with normal LV but also in those with LV dysfunction. This suggests that the response of exercise capacity to CR can be (at least partly) independent of LV function, and other determinants for exercise capacity also play important roles. We are fully aware that LV function must be involved in the effect of CR, so we analyzed the LV function data (data not shown) and did not find significant serial changes. Unfortunately, we failed to perform further analysis on parameters of diastolic function. In the present study, the enrolled patients were in a relatively well-compensated cardiac condition, and the LV function was assessed only by echocardiography at rest, which was a clear limitation. To clarify this point, examination of LV function during exercise should be undertaken, and a specific study is needed. In fact, it was recently reported that improvement in exercise capacity after CR of patients with preserved LVEF after AMI is associated with an increased heart rate response and better peripheral oxygen extraction during exercise [27]. Furthermore, the data on exercise capacity in the present study showed that phase II CR along with subsequent secondary prevention care preserved a long-term effect on physical performance in these patients. This is quite encouraging because previous studies have shown a remarkable loss of the effect on exercise capacity afterward [28].

The level of NT-proBNP is well recognized as a marker for the severity of myocardial injury and subsequent LV dysfunction [9] and is elevated early in ACS [29]. In the present study, the NT-proBNP level was clearly elevated before CR above the cutoff reference level (Figure 2(b)). As expected in the study, the patients with LV dysfunction had higher NT-proBNP levels than the patients with normal LV function. However, the level of NT-proBNP did not change significantly immediately after the CR program. To date, only a few studies have investigated the kinetics of BNP. Leroy et al., for instance, reported an early increase after the onset of ischemia (chest pain) and a peak at 14–24 hours and a progressive decrease in BNP thereafter [30]. A previous study showed that a three-month CR program based on physical exercise could accelerate the decrease in NT-proBNP levels compared to that without a CR program [24]. Two reasons may be responsible for this discrepancy between the previous studies and our current study. In the study of Giallauria et al., the level of NT-proBNP before CR was clearly higher (approximately 1500 pg/ml) than that of our study, suggesting different collectives of the subjects enrolled in the studies. The CR program performed in their study was three months, but the level of NT-proBNP after CR was still elevated (470 pg/ml). Both of these studies indicate a long halftime of NT-proBNP. Certainly, to clarify the effect of CR on NT-proBNP levels, a control group should be included in the study, which was not the major goal of the present study. The characteristic serial changes of NT-proBNP in the current study may imply that NT-proBNP alone seems unsuitable for assessing the acute beneficial effect of CR.

NT-proBNP levels at 12 months decreased significantly in patients with or without LV dysfunction in comparison with those before or after CR. This seemed to be accompanied by functional and clinical improvement. A previous study showed that the dynamic changes in NT-proBNP in patients with ACS decreased gradually for at least six months [9]. Factors associated with less reversibility of NT-proBNP are related to chronically impaired LV function [9]. This seems true in the present study since even at 12 months after CR, the NT-proBNP level (479 pg/ml) in patients with LV dysfunction remained 4-fold higher than that (121 pg/ml) in patients with preserved LV function. Unfortunately, we failed to perform further analysis on parameters of diastolic function.

A number of studies have shown that adiponectin levels are associated with the development and progression of coronary artery disease [14], and generally, an increase in adiponectin is beneficial for the outcome of the disease [13,14]. In the current study, the absolute value of adiponectin significantly decreased before CR (Figure 2(c)) but was higher than that reported previously [31], possibly due to different study populations and laboratory standards. Some factors could affect adiponectin levels; for instance, body mass index was negatively associated with adiponectin [32]. In the present study, the adiponectin level after CR, especially at the 12-month follow-up, increased, which is consistent with previous observations [33]. The elevated adiponectin level at the 12-month follow-up suggests a long-term beneficial effect of CR. It has been reported that body weight loss (>10%) after training was accompanied by an increase in adiponectin levels [34]; however, in the present study, no reduction in body weight occurred after CR, implying that the effect of CR on adiponectin levels was not due to changes in body weight. Unfortunately, we have not examined body composition, and thus, whether a change in adiponectin levels was associated with fat mass remains unclear.

Although previous studies have shown that CR improved exercise capacity and affected NT-proBNP and adiponectin levels [33, 35], no study dealing with all these parameters at different time points has been reported. The present study shows that among the changes in these parameters, there was no close correlation. This implies that the kinetics of the changes in these three parameters were different or not parallel, and the regulation of these parameters was independent. For instance, while NT-proBNP is mainly driven by LV function [9], exercise capacity in ACS patients is determined by both LV function and peripheral skeletal muscle [36]. In this context, in assessing and following up the outcome of an acute intervention or a CR program, all these parameters at different time points should be taken into consideration.

## 5. Conclusions

From acute care of an ACS through phase II CR to the 12-month follow-up, exercise capacity and blood levels of NT-proBNP and adiponectin undergo significant changes, but these changes are not parallel or not correlated with each other. Therefore, they provide complementary information from different aspects and should be taken together to evaluate the outcomes of ACS and CR.

## Figures and Tables

**Table 1:** Basic clinical data in patients before and after CR as well as at 12 months.

Basic characteristics			

Age (years)	56 ± 10		
M/F	69/12		
Diabetes mellitus	11 (13.6)		
Hypertension	60 (74.1)		
Hyperlipidemia	60 (74.1)		

*Coronary artery disease*			
1 vessel	27		
2 vessels	35		
3 vessels	19		
ST-elevation MI	68 (83.9)		
Non-ST-elevation MI	10 (12.3)		
Unstable angina	3 (3.7)		

Revascularization at 12-month follow-up			
Re-PCI/stent	18		
CABG	3		

	Before CR	After CR	12-month
*Risk factors*			
Smoking	66 (81.5)	0 (0.0)^a^	15 (18.5)^b,c^
Weight (kg)	85.4 ± 14.7	84.6 ± 14.0	87.1 ± 15.2^b,c^
BMI (kg/m^2^)	27.8 ± 3.6	27.5 ± 3.5	28.6 ± 3.8^b,c^
Cholesterol (mmol/l)	5.1 (4.3–6.1)	4.3 ± 1.2^a^	4.5 ± 1.0^b^
HDL (mmol/l)	1.2 ± 0.3	1.3 ± 0.4	1.2 ± 0.3
LDL (mmol/l)	2.4 ± 0.8	2.3 ± 0.8	2.5 ± 0.8
Triglycerides (mmol/l)	2.0 (1.1–2.9)	1.5 (0.8–2.6)	1.5 (1.0–2.4)
Glucose (mg/dl)	89 (72–115)	87 (81–102)	94 (81–107)
CRP (*µ*g/ml)	12.96 (7.25–18.76)	2.04 (0.49–5.61)^a^	2.28 (0.42–4.37)^b^
Creatinine (*µ*mol/l)	96 (84–111)	94 (87–141)	94 (88–113)

*Medication*			
Aspirin	79 (97.5)	80 (98.8)	79 (97.5)
ACE inhibitor	71 (87.7)	68 (84.0)	58 (71.6)^b^
Beta-blocker	79 (97.5)	77 (95.1)	69 (85.2)^b,c^
Ca^2+^ antagonist	3 (3.7)	3 (3.7)	4 (4.9)
Statin	77 (95.1)	79 (97.5)	66 (81.5)^b,c^

*LV dysfunction*			
EF < 50%	27 (33.3)	32 (39.5)	16 (19.8)^c^
Mild: EF 41–50%	18 (22.2)	23 (28.4)	12 (14.8)^c^
Moderate: EF 30–40%	8 (9.9)	8 (9.9)	1 (1.2)^b,c^
Severe: EF <30%	1 (1.2)	1 (1.2)	3 (3.7)
*Quality of life*			
Scores	70 (50–80)	80 (67–90)^a^	80 (60–90)^b^

Data are presented as mean ± SD or median (interquartile) or number (%) of patients. ^a,b^Compared to before CR, *P* < 0.05; ^c^Compared to after CR, *P* < 0.05; MI, myocardial infarction; PCI, percutaneous coronary intervention; CABG, coronary-artery-bypass graph; LV, left ventricle; EF, ejection fraction; CR, cardiac rehabilitation; BMI, body mass index; HDL, high-density lipoprotein cholesterol; LDL, low-density lipoprotein cholesterol; CRP, C-reactive protein; ACE, angiotensin converting enzyme.

## Data Availability

The data used to support the findings of this study are available from the first and corresponding authors upon request.
